# Characterisation of Potential Antidiabetic-Related Proteins from *Pleurotus pulmonarius* (Fr.) Quél. (Grey Oyster Mushroom) by MALDI-TOF/TOF Mass Spectrometry

**DOI:** 10.1155/2014/131607

**Published:** 2014-08-28

**Authors:** Nurul Azwa Abd. Wahab, Noorlidah Abdullah, Norhaniza Aminudin

**Affiliations:** ^1^Mushroom Research Centre, Institute of Biological Sciences, Faculty of Science, University of Malaya, 50603 Kuala Lumpur, Malaysia; ^2^University of Malaya Centre for Proteomics Research, University of Malaya, 50603 Kuala Lumpur, Malaysia

## Abstract

*Pleurotus pulmonarius* has been reported to have a potent remedial effect on diabetic property and considered to be an alternative for type 2 diabetes mellitus treatment. This study aimed to investigate the antidiabetic properties of ammonium sulphate precipitated protein fractions from *P. pulmonarius* basidiocarps. Preliminary results demonstrated that 30% (NH_4_)_2_SO_4_ precipitated fraction (F30) inhibited *Saccharomyces cerevisiae* α-glucosidase activity (24.18%), and 100% (NH_4_)_2_SO_4_ precipitated fraction (F100) inhibited porcine pancreatic α-amylase activity (41.80%). Following RP-HPLC purification, peak 3 from F30 fraction demonstrated inhibition towards α-glucosidase at the same time with meagre inhibition towards α-amylase activity. Characterisation of proteins using MALDI-TOF/TOF MS demonstrated the presence of four different proteins, which could be implicated in the regulation of blood glucose level via various mechanisms. Therefore, this study revealed the presence of four antidiabetic-related proteins which are profilin-like protein, glyceraldehyde-3-phosphate dehydrogenase-like protein, trehalose phosphorylase-like (TP-like) protein, and catalase-like protein. Hence, *P. pulmonarius* basidiocarps have high potential in lowering blood glucose level, reducing insulin resistance and vascular complications.

## 1. Introduction

Diabetes mellitus (DM) is a chronic metabolic disorder in the endocrine system resulting from defects of insulin secretion (type 1), increased cellular resistance to insulin (type 2), or both. The consequence of this is characterised by an abnormally high level of blood glucose, also known as hyperglycemia, that leads to serious damage of the body organs [1]. Without sufficient insulin availability, body tissues, particularly the liver, muscle, and adipose tissues will fail to take and utilise glucose from the blood circulation. DM is a heterogeneous disease with both genetic and environmental causative factors such as lack of physical exercise. The genetic factor, however, has always been the major factor in the development of diabetes [2].

DM is rapidly increasing worldwide and has currently become the third “killer” of human beings. Statistics have shown that type 2 diabetes is likely to increase from 150 million to 225 million by the end of the decade and is expected to increase further to 300 million by 2025 [3]. Type 2 diabetes is the most prevalent form of diabetes compared with type 1 and gestational diabetes. It remains undiagnosed until now and accounts for 90–95% of all diagnosed diabetes cases [4]. Uncontrolled DM disease leads to the development of both acute and chronic complications, such as retinopathy, neuropathy, amputation, organ dysfunction involving the eyes, kidneys, nervous system, and heart, and damage of vascular systems, thereby increasing the risk of cardiovascular disease [5]. Moreover, untreated complications resulting from this disease may eventually lead to death [6].

Currently, several DM therapeutic drugs are available in the market. This includes various oral antidiabetic agents such as sulfonylureas, biguanides, glinides, tolbutamide, phenformin, troglitazone, rosiglitazone, and repaglinide. Even though there are many drugs available, most of them are too toxic and costly and promote negative effects on the patient. Thus, they significantly fail to alter the course of diabetic complications. Some of these drugs may potentially increase the incidence of renal tumours, hepatic injury, and acute hepatitis [7]. Currently, most antidiabetic researches focused highly on the development of antihyperglycemic agents that are safe and free of adverse effects such as nausea, diarrhoea, liver problems, and weight gain [8].

It has been recently reported that many researchers are working on an alternative therapeutic approach for combating DM. Studies aimed at decreasing postprandial hyperglycemia by delaying the absorption of carbohydrates/glucose through inhibition of carbohydrate hydrolysing enzymes, α-amylase, and α-glucosidase using natural sources such as various species of plants and fungi by bioassay-guided fractionation [9].

In this study, potential bioactive proteins will be isolated and characterised via proteomics approach, which is an excellent tool that enables profiling, discovering, and identifying proteins [10]. Bioactive proteins are the major focus of this study because of the abundance and wide variety of proteins that had been reported to possess interesting biological actions and applicable activities [11]. Proteome analyses involved several methods and protein isolation techniques such as ammonium sulphate precipitation, high-performance liquid chromatography (HPLC), SDS-PAGE, and MALDI-TOF/TOF mass spectrometry [12].

A wide variety of mushroom species were found to be effective for controlling the blood glucose levels and modifying the course of diabetic complications. One of the excellent mushroom candidates speculated to have these antidiabetic properties is* Pleurotus pulmonarius* (grey oyster mushroom). Bioactive compounds from mushrooms and other natural sources are considered to be less toxic and free from side effects compared with synthetic drugs. Biologically active compounds that have been extracted from mushrooms include polysaccharides, proteins, and dietary fibres, isolated whether from whole mushroom or mycelia [13].

The production of* P. pulmonarius* has been rapidly increasing worldwide because of its broad adaptability, aggressiveness, ease of cultivation with high yield potential, and high nutritional value. Furthermore, it can be cultivated within a wide range of temperatures on different natural resources and agricultural wastes [14]. Previous research reported that* P. pulmonarius *has a good potential in reducing hyperglycemia in type 2 diabetes and ameliorates the course of cardiovascular-related complications [15]. The aqueous extract of* P. pulmonarius* basidiocarps contains vitamins B1, B2, and C, carbohydrate, calcium, and low calories. This mushroom also has the potential to reduce cholesterol level in the blood [16]. According to* Icons of Medicinal Fungi from China*, the medicinal part of the oyster mushroom is in its basidiocarps [17, 18].

To date, there was no report on the active protein(s) of antidiabetic properties isolated from the* P. pulmonarius* basidiocarps. Hence, the present study aims to isolate, identify, and characterise potential bioactive proteins with antidiabetic activity from* P. pulmonarius *basidiocarps.

## 2. Materials and Methods 

### 2.1. Preparation of* P. pulmonarius* Samples

Basidiocarps of* P. pulmonarius* were purchased from a local hypermarket; it was cleaned and cleaved into small pieces. Basidiocarps were frozen at −20°C prior to freeze-drying. Freeze-dried mushroom basidiocarps were then ground using a dry grinder to obtain fine powder, and the samples were kept in a dry container at room temperature prior to extraction procedure.

### 2.2. Preparation of* P. pulmonarius *Aqueous Extract

Freeze-dried* P. pulmonarius *powder (30 g) was weighed and soaked in 300 mL of distilled water (ratio 1 : 10). The mixture was then homogenised, and the homogenate was separated by filtration using a vacuum pump. The filtrate was collected and spun at 5,000 rpm for 20 minutes at 4°C. The supernatant was then stored at −20°C. Protein content was estimated using BCA Protein Assay Kit (Pierce).

### 2.3. Fractionation of Proteins Using Ammonium Sulphate Precipitation Method

A series of protein fractions was obtained by subjecting the aqueous extracts to ammonium sulphate ((NH_4_)_2_SO_4_) precipitation. Sufficient amount of solid (NH_4_)_2_SO_4_ to form 10% to 100% salt saturation solutions was prepared. (NH_4_)_2_SO_4_ was slowly added with continuous stirring. The solution was then centrifuged at 10,000 rpm for 30 minutes (4°C). Protein was recovered by resuspending the precipitate gently in 1 mL of distilled water and then dialyzed using SnakeSkin pleated dialysis tubing with 3,500 Da molecular weight cut-off (Thermo Scientific). The dialysis process was done at 4°C for 48 hours with 4 changes of distilled water. The dialysate was freeze-dried, stored at −20°C, and referred to as (NH_4_)_2_SO_4_ protein fraction (F10–F100).

### 2.4. Inhibition Assay for α-Glucosidase Activity

The determination of α-glucosidase inhibitory activity was performed by the spectrophotometric method according to Apostolidis et al. [19], with modification. This assay was carried out in the dark as* p*-nitrophenyl-D-glucopyranoside, which is light sensitive. Protein content was determined using BCA Protein Assay Kit (Pierce).

A volume of 250 *μ*L of F1–F100 solution and 250 *μ*L of 1.0 M potassium phosphate buffer (pH 6.9) containing* Saccharomyces cerevisiae*  α-glucosidase solution (0.1 U/mL) were premixed in a test tube and then preincubated at 37°C for 2 minutes. Following preincubation, 250 *μ*L of* p*-nitrophenyl-D-glucopyranoside in 1.0 M phosphate buffer (pH 6.9) was added and further incubated at 37°C for 30 minutes. The catalytic reaction was terminated by the addition of 2 mL 0.1 M sodium carbonate (Na_2_CO_3_) solution. Absorbance was recorded at 405 nm by Shimadzu UV Mini 1240 spectrophotometer and compared to a control, consisting of 250 *μ*L of distilled water in place of the protein fraction. Voglibose was used as a positive control. The α-glucosidase inhibitory activity was expressed as inhibition % and was calculated as follows:

(1) Percentage  of  Inhibition  =[(A405Control−  A405Extract)]×100A405Control

A_405_Extract is OD 405 value with protein fraction,* p*-nitrophenyl-α-D-glucopyranoside, and α-glucosidase.

A_405_Control is OD 405 value with* p*-nitrophenyl-α-D-glucopyranoside and α-glucosidase.

### 2.5. Inhibition Assay for α-Amylase Activity

The α-amylase inhibition assay was adapted from Apostolidis et al. [20] with some modifications. A volume of 1 mL of protein extracts (25 *μ*g/mL) was premixed in 1 mL of 0.02 M sodium phosphate buffer (pH 6.9 with 0.006 M sodium chloride) containing 0.5 mg/mL porcine α-amylase, which was incubated at 37°C for 5 minutes. Following preincubation, 1 mL of a starch solution in 0.02 M sodium phosphate buffer (pH 6.9 with 0.006 M sodium chloride) was added to each tube and the reaction mixtures were then incubated again at 37°C for 10 minutes. The reaction was ended by directly transferring the reaction mixture to a boiling water bath (100°C), and 2 mL of dinitrosalicylic acid (DNS) colour reagent was quickly added while boiling and left for 15 minutes. The reaction mixture was cooled to room temperature and diluted by adding 5 mL of distilled water. Absorbance was measured at 540 nm. Acarbose was used as a positive control. The α-amylase inhibitory activity was expressed as inhibition % and was calculated as follows:

(2) Percentage  of  Inhibition  =[(A540Control−  A540Etract)]×100A540Control

A_540_Extract is OD 540 value with protein fraction, starch, and α-amylase enzyme.

A_540_Control is OD 405 value with starch and α-amylase.

### 2.6. Purification of Active Protein Fractions by Reversed Phase: High-Performance Liquid Chromatography (RP-HPLC) Analysis

Active protein fractions for both antidiabetic assays were further purified by RP-HPLC (Shimadzu, Japan). Analytical Atlantis T3 C_18_ Column (4.6 mm × 250 mm × 5 *μ*m) was used for protein separation. Fractions were eluted using binary gradient of acetonitrile (0–80%, v/v) and 0.1% TFA at flow rate 1 mL/min for 40 minutes. However, for sample collection, Chromolith SemiPrep RP-18 endcapped 100–10 mm was used to collect eluted sample using the same developed method. Elution was done at the flow rate of 3.9 mL/min, with UV detector set at 220 nm. Eluted RP-HPLC protein fractions were exposed to a stream of nitrogen gas to remove the acetonitrile and later freeze-dried. Antidiabetic activity of the fractions collected from RP-HPLC was then evaluated by the same* in vitro* antidiabetic assays.

### 2.7. Profiling of Active Protein Fractions Using Sodium Dodecyl Sulfate Polyacrylamide Gel Electrophoresis (SDS-PAGE)

Active RP-HPLC protein fractions were subjected to SDS-PAGE using Laemmli method [21], prior to MALDI-TOF/TOF MS analysis. The protein separation was done on 16% polyacrylamide Tris/HCl gels. Prestained Protein Marker, broad range 6–175 kDa (Bio-Rad), was used as a molecular weight marker. Samples were boiled at 90°C for 5 minutes prior to electrophoresis. The electrophoresis was carried out using Bio-Rad Power Pac 300 at a constant voltage of 60 V for the first 15 minutes, followed by gradual increase to 80–100 V. The gels were then stained with Coomassie Brilliant Blue R-250 and documented. In order to improve visualisation of faint protein bands, MS compatible silver staining method was employed. The gels' images were captured for identifications. Excised protein bands from silver-stained SDS-PAGE gel were subjected to in-gel tryptic digestion and later prepared for MALDI-TOF/TOF MS analysis. Intact RP-HPLC protein fractions were also subjected to in-solution tryptic digestion and MALDI-TOF/TOF MS.

### 2.8. Protein Identification by MALDI-TOF/TOF Mass Spectrometry

The protocol used for in-solution tryptic digestion was modified from Pierce In-solution Tryptic Digestion Kit. Proteins were resuspended in 15 *μ*L of digestion buffer and 1.5 *μ*L of reducing buffer. This mixture was incubated at 95°C for 5 minutes. Alkylation buffer was added and incubated in the dark for 20 minutes. In order to digest the sample, 2 *µ*L of trypsin was added and incubated overnight at 37°C. The mixture was shook with 50 *μ*L of 50% acetonitrile for 15 minutes to extract the proteins. All liquid was transferred to fresh tubes. The method was repeated by adding 100% acetonitrile for 15 minutes. The digested samples were then dried completely by using vacuum at low speed. The samples were desalted using a Zip Tip (C_18_) (Millipore), directly spotted onto the sample plate, and analysed using 4800 Plus MALDI-TOF/TOF MS (Applied Biosystems). Analysis was done to match known proteins or translated open frames in the database of National Centre for Biotechnology Information (NCBI) and Swissprot.

For in-gel tryptic digestion, excised protein bands were sent to Genome Research Centre, the University of Hong Kong (HKU), and analysed using ABSciex MALDI 4800 TOF/TOF Analyzer. The scanning range for general protein identification was 900–4,000 m/z, in reflector mode. Five of the most abundant peptides (precursors) that are not on the exclusion list were selected for further fragmentation (MALDI-TOF/TOF) analysis to generate the full scan mass spectrum. MASCOT (Ver. 2.1) and NCBI search analysis settings were as follows: fixed modification: carbamidomethyl (C), variable modification: oxidation (M), MSMS fragment tolerance: 0.2 Da, precursor tolerance: 75 ppm, and peptide charge: +1, monoisotopic.

### 2.9. Statistical Analysis

All assays were performed in triplicates, and the values were averaged; each data expressed represents the mean ± standard deviation (SD). Data significance was performed by one-way ANOVA by Minitab statistical software program version (14.12.0). Differences of 95% (*P* < 0.05) were considered statistically significant.

## 3. Results

### 3.1. *In Vitro* Screening of* P. pulmonarius* Aqueous Extract and Protein Fractions for Inhibition of α-Glucosidase and α-Amylase Activities

**Figure 1:**
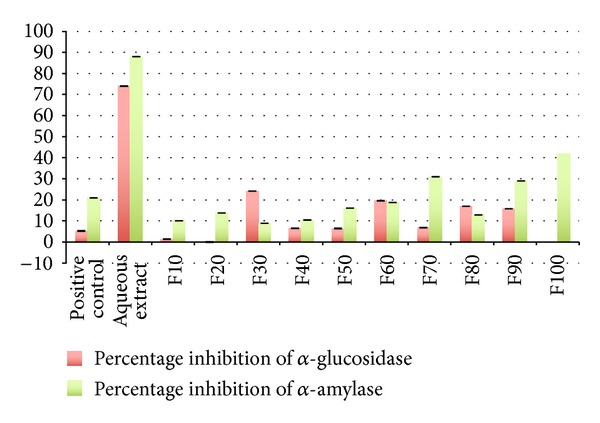
Percentage inhibition for α-glucosidase and α-amylase enzymes for F10–F100 at 25 *μ*g/mL concentration and aqueous extract at 25 mg/mL. α-Amylase enzyme is more susceptible to inhibition compared to α-glucosidase enzyme. The positive controls used were voglibose for α-glucosidase and acarbose for α-amylase.

(NH_4_)_2_SO_4_ precipitated protein fractions (F10–F100) were assayed for their inhibitory activities against α-glucosidase and α-amylase enzymes. For both assays, the protein concentration in each function was fixed at 25 *μ*g/mL. F30 suppressed the activity of α-glucosidase enzyme (24.18%), while F100 showed the highest percentage inhibition activity for α-amylase enzyme (41.80%) (Figure 1).

### 3.2. Purification of Active (NH_4_)_2_SO_4_ Protein Fractions by Reverse-Phase High-Performance Liquid Chromatography (RP-HPLC)

**Figure 2:**
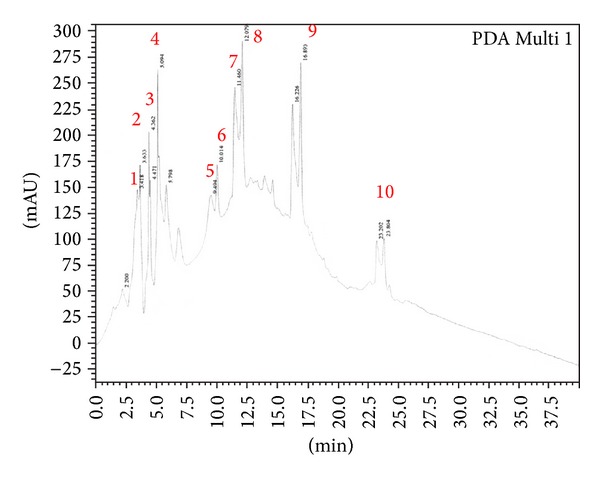
RP-HPLC profile of F30 fraction. Detection was done at wavelength of 220 nm.

**Figure 3:**
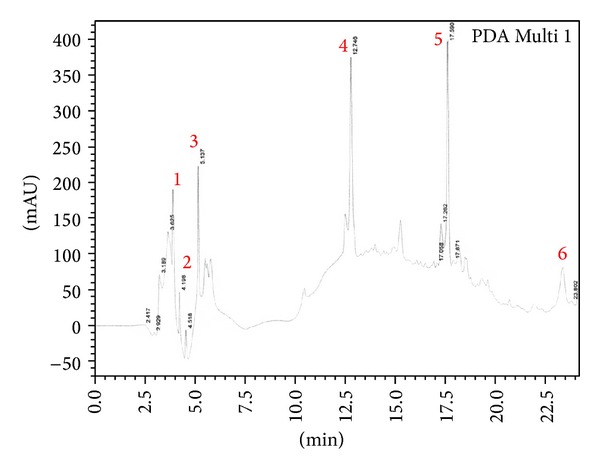
RP-HPLC profile of F100 fraction. Detection was done at wavelength 220 nm.

F30 fraction that showed the highest inhibition towards α-glucosidase was selected to be further purified by RP-HPLC. Distinct peaks were collected; however, among all the RP-HPLC peaks, peak 3 (Figure 2) strongly suppressed the activity of α-glucosidase enzyme (25%) at 2.5 *µ*g/mL concentration. On the other hand, F100 fraction that showed the highest inhibition towards α-amylase was selected for further purification by RP-HPLC. However, F100 fraction (Figure 3) at 6.25 *µ*g/mL only showed 2.85% of inhibition towards the activity of α-amylase (Table 1). In view of this data, all fractions of F100 eluted from the RP-HPLC (peak 1–6) were combined and evaluated for α-amylase activity. This combined F100 (CF100) showed 12% inhibition towards the enzyme, higher than the inhibition shown by individual peaks.

Percentage inhibition of α-glucosidase and α-amylase enzymes at 2.5 *µ*g/mL and 6.25 *µ*g/mL RP-HPLC eluted peaks concentrations, respectively. CF100 is the combination of all eluted peaks from fraction F100. The positive controls used were voglibose for α-glucosidase and acarbose for α-amylase. The data shown are means of three replicates.

Following the separation of F30 fraction by RP-HPLC, eluted peaks were collected and indicated by numbers 1–10. All peaks were tested for α-glucosidase assay* in vitro*.

Following the separation of F100 fraction by RP-HPLC, eluted peaks were collected and indicated by numbers 1–6. All peaks were tested for α-amylase assay* in vitro*.

### 3.3. Profiling of Peak 3 from F30 Fraction Using Sodium Dodecyl Sulfate-Polyacrylamide Gel Electrophoresis (SDS-PAGE)

**Figure 4:**
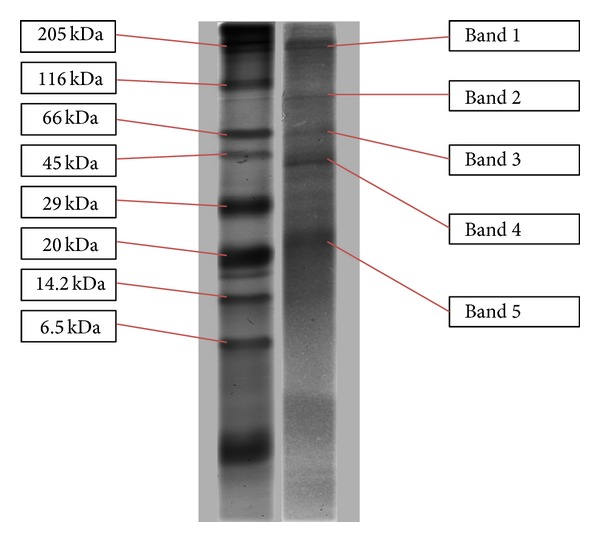
Silver-stained gel image of active peak 3 from F30 fraction. Lane 1: prestained SDS-PAGE standards protein marker (6–175 kDa) (Bio-Rad). Lane 2: peak 3 from F30 fraction; 5 bands were resolved following electrophoresis (bands 1–5).

SDS-PAGE profile of the active RP-HPLC peak 3 from F30 (Figure 4) showed the presence of five distinct protein bands. From the SDS-PAGE, recognised bands were excised and prepared for further identification by MALDI-TOF/TOF MS analysis.

### 3.4. Identification of Proteins by MALDI-TOF/TOF MS Analysis 

Identification of proteins in peak 3 from F30 fractions was done using two approaches, in-gel and in-solution tryptic digestions, followed by procedures for MALDI-TOF/TOF MS analysis. For the in-gel one, protein bands observed by SDS-PAGE profile were excised, digested, and analysed, while, for the in-solution one, protein solution of peak 3 from F30 fraction was directly digested and analysed. Proteins identified from the MS analysis of in-gel tryptic digestion method are profilin-1:* Phaseolus vulgaris*, profilin-1:* Ricinus communis*, and profilin-1:* Glycine max* while for in-solution tryptic digestion showed the presence of trehalose phosphorylase, glyceraldehyde-3-phosphate dehydrogenase (GAPDH), HSP 100, WUN, RNA polymerase II second largest subunit, antimicrobial ribonuclease, heat-induced catalase, laccase 3, translation elongation factor 1 alpha, catalase, and laccase 4. All identified proteins were further researched against several reported literatures regarding their biological importance. Among those identified proteins, four proteins showed possible relationship with antidiabetic mechanism directly or indirectly. Those proteins were listed in Table 2.

Table 2 shows the possible antidiabetic-related proteins obtained from MALDI-TOF/TOF MS. Protein search was done using NCBI and MASCOT database for* Lentinus* species.

## 4. Discussion

This study demonstrated the* in vitro* antidiabetic activity of* P. pulmonarius* basidiocarps protein fractions based on two assays involving carbohydrate metabolizing enzymes: α-glucosidase and α-amylase. The inhibition of these enzymes, which were involved in the digestion of carbohydrates, can significantly reduce postprandial elevations of blood glucose and, therefore, may serve as alternative strategy in the management of blood glucose level especially in type 2 DM [22].* P. pulmonarius* basidiocarps have long been regarded as useful in disease prevention and treatment because of their low fat and high-soluble fibre content [23].

For α-glucosidase enzyme activity, F30 protein fraction suppressed the activity of the enzyme at 24.18%. Meanwhile, F100 protein fraction showed 41.80% inhibition of α-amylase enzyme. These results suggested the involvement of different types of proteins for respective enzymes. Based on the amount of salt saturation required for their precipitation (30% and 100%), it could be hypothesised that these proteins have different hydrophobic strengths with F30 fraction being more hydrophobic; meanwhile, F100 fraction is very much hydrophilic [24]. Hydrophobicity (and hydrophilicity) had been shown to have a major role in the binding behaviour of specific drugs and proteins [25]. This could explain how the enzymes work at the different places in human intestinal tract and with different mechanisms of action. According to Melander [26], the hydrophobicity characteristic explains the differences in clinical efficacy or safety of therapeutic drugs even though it shows identical mechanisms of action. Examples of hydrophobic antidiabetic drugs are sulfonylureas, phenformin, and adenosine monophosphate-activated protein kinase (AMPK) [27]; meanwhile, an example for hydrophilic is metformin [28].

Distinct peaks from RP-HPLC were collected and further examined with bioassay-guided assays. Peak 3 from F30 fraction exerted significant inhibitory activity (25%) towards α-glucosidase at the concentration of 2.5 *µ*g/mL. This value is close to the inhibition percentage given by the F30 fraction (24.18%) at a concentration of 25 *µ*g/mL, (10x higher). This observation suggests that peak 3 from F30 fraction could contain the bioactive protein that gives the inhibitory effect. Meanwhile, peak 3 from F100 fraction demonstrated the highest percentage of inhibition towards α-amylase at only 2.84%. This is a tremendous decrease compared with 48.1% inhibition showed earlier by F100 fraction. All eluted peaks from RP-HPLC of F100 were then recombined, and inhibition rates were determined. Combined fraction (CF100) demonstrated an increase to 12% of inhibition. These results indicated a possible synergistic interaction between proteins in F100 fraction because they effectively inhibited α-amylase activity when present as a group or mixture, but the inhibition effect became insignificant once they were separated through RP-HPLC [29, 30]. CF100 did not manage to achieve the original inhibition percentage of F100, possibly because of the absence of uncollected eluted fraction and/or alteration in the binding formation between the collected and uncollected proteins in the eluted fraction. This suggests that purification lowers the inhibition activity, thus implicating the synergistic interaction.

Peak 3 from F30 fraction was subjected to SDS-PAGE prior to in-gel digestion for MALDI-TOF/TOF MS analysis, and 5 distinct protein bands (Figure 4) were observed. Mass spectrometry analysis against* Lentinus* NCBI database revealed the presence of several proteins with different roles, functions, and involvement in biological systems. However, 3 proteins, which include GAPDH, catalase, and trehalose phosphorylase, were of interest in this study. These identified proteins have a possible relationship with antidiabetic activity and involved in the regulation of blood glucose level through different mode of actions.* Lentinus* NCBI was selected in detecting proteins because it is the closest genus to mushroom.

On the other hand, for in-solution MALDI-TOF/TOF MS, using MASCOT database, profilin was the most abundant protein detected and has a potential mechanism in inhibiting diabetes as well. The identification of profilin from in-solution mixture could be attributed to its small molecular weight. Profilin could have diffused out from the SDS-PAGE gel and thus not detected in in-gel tryptic digestion MS analysis.

Profilin (14 kDa) is an enzyme commonly found in various eukaryotes' organism ranging from fungi to animal [31]. In diacylglycerol-protein kinase C pathway (DAG-PKC), phosphatidylinositol 4,5-bisphosphate (PIP_2_) is hydrolysed by phospholipid C (PLC) into 2 signalling molecules, diacylglycerol (DAG) and inositol trisphosphate (IP_3_). IP_3_ opens calcium channel to allow calcium entry from endoplasmic reticulum and extracellular fluid. An increase in cellular calcium encouraged DAG to activate the protein kinase C (PKC) [32]. The activation of PKC could result in the complication of DM because it enhances contractibility, permeability, and vascular cell proliferation. It is also associated with neurovascular changes, insulin resistance, and retinal abnormalities [33]. Moreover, in diabetic cases, the augmented availability of glucose causes an augmented availability of DAG, which activates PKC [34].

From previous research, profilin works as a negative regulator of PLC, implicated thus in the inhibition of PKC activation. Profilin binds with high affinity to PIP_2_ and interferes and lowers the rate of PIP_2_ hydrolysis by PLC, thereby reducing the formation of IP_3_ and DAG [35]. Inhibition of PKC activation can prevent the progression of DM and blocks the potential of vascular complications [36]. This finding revealed that profilin-like protein might play a critical role and represent a promising therapeutic target in preventing DM as well as becoming a mediator in diabetic complications. The results from our study demonstrated the potential inhibition of peak 3 from F30; this suggests that this peak probably contains a protein that is similar to the structure or characteristic of profilin. Currently, the PKC inhibitor drugs available in the market are toxic; therefore, profilin-like protein extracted from* P. pulmonarius* could be a starting point to an alternative treatment for DM. Drug development in this area is important because it not only offers new treatment strategy for DM but also contributes to therapeutic options for cancer, autoimmune disease, and inflammation because it also acts as a tumour suppressor in human carcinomas [37].

The discovery of GAPDH-like protein (37 kDa) adds another possibility in combating DM disease [38]. GAPHD oxidatively phosphorylates glyceraldehyde-3-phosphate (GAP) to 1,3-diphosphoglycerate which is considered to provide a common link between hyperglycemia-induced, oxidative stress, and activation pathways associated with DM complication [39].

In most diabetic cases, hyperglycemic condition tends to increase enzymatic conversion of glucose to polyalcohol sorbitol, reduces NADPH and glutathione source. Since the conversion of sorbitol to fructose requires NAD, it would reduce the amount of nucleotide available for GAPDH activity. Inhibition of GAPDH involves the formation of methylglyoxal (MG) and advanced glycation end products (AGEs), which contribute to the development of diabetic complication [40].

MG is the most powerful nonenzymatic glycating agent found in diabetic patients, which give rises to vascular complication. The toxic potential of MG effects insulin secretion and acts as a major precursor for AGE formation. In hyperglycemia, AGEs glycate with protein, which results in saturation that will cause loss of functions and poor gene expression. AGEs cause plaque formation in blood vessel, leading to high blood pressure, amputation, and cataracts. Indeed, both MG and AGEs can induce oxidative stress, inflammation, and mitochondrial dysfunction, which is implicated in DM complication and effects neuropathological changes in the brain [41].

GAPDH production could be modified by environmental factors or genetic dysregulation, which then affects MG and AGEs production [42]. However, GAPDH gene expression can be regulated by hormonal, nutritional, and metabolic factors including insulin, which can increase GAPDH mRNA and activity. Therefore, GAPDH could contribute towards the control of crucial metabolic step that determines the levels of MG precursor and regulates AGEs formation in the body system. The actual amount and function of GAPDH-like protein in* P. pulmonarius* are not clear at this point, but its presence could be an important key to the control of metabolic processes that may have a certain relationship with diabetics.

The third protein identified in this study is TP-like protein (84 kDa). TP is an enzyme, widely found in many microorganisms, fungi, plants, and animals [43]. It reversibly catalyzes trehalose synthesis via degradation of α-glucose-1-phosphate (α-Glc-1-P) and glucose. TP plays a vital role in trehalose, starch, sucrose, and glycogen metabolisms [44].

The commercial production of trehalose as a protein food as a replacement of sucrose especially for DM patients developed in a large scale in 1995 [45]. Other than that it helps in inhibiting progression of type 2 DM and the intake of trehalose has been shown to give better effects such as evoking lower insulin secretion, mitigating insulin resistance, and reducing osteoporosis development [46].

In addition, trehalose and TP are both involved either in the production or in degradation of glycogen. TP affects the levels of glycogen and utilisation of trehalose in cells by catalysing the conversion of glycogen to trehalose when cytoplasmic trehalose levels are depleted and vice versa. It could be concluded that TP not only is involved in the production of trehalose from glycogen but also appears to be important in the formation and accumulation of glycogen [47].

In summary, it is good for a diabetic patient to replace the intake of sugar (sucrose) with trehalose, owing to the ability of TP to level off trehalose in the body by converting it to glycogen. Removal of excessive trehalose from the body is probably essential because high levels of trehalose may also be toxic. Therefore, the presence of TP-like protein in* P. pulmonarius* could act as a regulatory molecule in the control of trehalose metabolism to glucose transport and glycolysis. TP controls intracellular levels of trehalose by converting the excessive trehalose to maltose, which can be further converted by α-glucosidases to glucose [48].

The forth protein is catalase enzyme (43 kDa), which catalyzes the breakdown of hydrogen peroxide (HP) into oxygen and water [49]. Catalase helps enhance the insulin secretion and sensitization. Other than controlling HP concentration, catalase will increase in response to oxidative stress and protect pancreatic beta cells from damage.

Deficiency of catalase (acatalasemia) may result in elevation of HP concentration and favour oxidative stress and contribute to late onset disorder such as DM. Surplus level of HP may cause toxic effect to proteins, RNA, DNA, and lipids and usually damages the pancreatic cells and inhibits insulin signalling [50]. A prolonged acatalasemia situation will result in homozygous mutations gene, and this will spark links with diabetes, Alzheimer's disease, and tumours [51].

Furthermore, high free radicals and low antioxidant mechanism will mitigate insulin resistance and damage cellular organelles and enzymes [52]. In type 2 diabetic patients, the downregulation of catalase synthesis may result in decreased blood catalase activity, thereby increasing HP production [53]. Therefore, it is necessary to take the right amount of minerals and proteins, such as barley grass, wheat, green sprouts, fruits, and vegetables, in order to sustain the sources of catalase in the body [54]. Because* P. pulmonarius* also contains catalase-like protein, consumption of this mushroom as a dietary component would help increase the blood catalase, thus helping in combating DM and complications. Currently, treatment of antioxidant-based drug formulations due to catalase gene alterations by captopril, aminoguanidine, melatonin, and acetylsalicylic acid is available but accompanied by several reported side effects [55].

## 5. Conclusion

Proteins derived from* P. pulmonarius* basidiocarps have a significant potential as alternatives in treating type 2 DM based on their ability to inhibit antidiabetic activity* in vitro*. These proteins include profilin-like protein, GAP dehydrogenase-like protein, TP-like protein, and catalase-like protein. However, at this point, the actual mechanism of action for these proteins remains unknown, and further in-depth* in vitro* as well as* in vivo* study could provide an explanation and validation.

## Figures and Tables

**Table 1:** Percentage inhibition of *α*-glucosidase and *α*-amylase enzymes.

Peaks from RP-HPLC and F100 fraction	Percentage inhibition of *α*-glucosidase by peaks from F30 fraction	Percentage inhibition of *α*-amylase by peaks from F100 fraction
Positive control	13 ± 2.03	2.10 ± 0.41
Peak 1	17 ± 0.70	0
Peak 2	18 ± 1.11	0
Peak 3	25 ± 2.57	2.85 ± 0.36
Peak 4	22 ± 2.99	1.11 ± 0.25
Peak 5	17 ± 0.98	1.44 ± 0.23
Peak 6	19 ± 2.37	0.11 ± 0.25
Peak 7	14 ± 0.72	—
Peak 8	22 ± 2.07	—
Peak 9	18 ± 1.20	—
Peak 10	17 ± 1.17	—
CF100	—	12 ± 5.20

**Table 2:** Antidiabetic-related proteins characterised based on NCBI and MASCOT database.

Name of protein	Accession number	Molecular weight	Protein score	Number of peptide matches	SDS-PAGE Band number
Identified proteins: searched against *Lentinus*; NCBI database					
Glyceraldehyde-3 phosphate dehydrogenase (GAPDH)	gi/30580405	36	13	2	2
Catalase	gi/28558774	43	9	3	5
Trehalose phosphorylase	gi/74626081	84	32	1	4

Identified proteins: searched against MASCOT database					
Profilin-1: *Phaseolus vulgaris* (kidney bean) (French bean)	PROF1_PHAVU	14 172	42	2	—
Profilin-1: *Ricinus communis* (castor bean)	PROF1_RICCO	14 199	42	2	—
Profilin-1: (GmPRO1) (Allergen Gly) glycine max (soybean)	PROF1_SOYBN	14 091	42	2	—
